# Different Coordination Modes of a Tripod Phosphine in Gold(I) and Silver(I) Complexes

**DOI:** 10.1155/MBD.1999.211

**Published:** 1999

**Authors:** P. Sevillano, M. E. García, A. Habtemariam, S. Parsons, P. J. Sadler

**Affiliations:** 1 Department of Inorganic Chemistry University of Santiago de Compostela Santiago de Compostela E-15706 Spain; 2 Department of Chemistry University of Edinburgh King's Buildings West Mains Road Edinburgh EH9 3JJ UK

## Abstract

The following gold(I) and silver(I) complexes of the tritertiary phosphine 1,1,1- tris(diphenylphosphinomethyl)ethane, *tripod* , have been synthesised: Au_3_(tripod)X_3_ [X = Cl(**1**), Br(**2**), I(**3**)]; [Au_3_(tripod)_2_Cl_2_]Cl (**4**); Au(tripod)X [X = Br(**5**), I(**6**)]; Ag_3_(tripod) (NO_3_)_4_ (**7**), Ag(tripod)NO_3_ (**8**). They were characterized by X-ray diffraction (complexes **2**, **3** and **4**), ^31^P NMR spectroscopy, electrospray and FAB mass spectrometry and infrared spectroscopy. Complexes **2** and **3** show a linear coordination geometry for Au(I), with relatively short Au-P bond distances. Complex **3** has a Au•••Au intramolecular distance of 3.326 $\overset{\text{°}}{\text{A}}$ , while complex **2** had a short Au•••Au intermolecular interaction of 3.048 $\overset{\text{°}}{\text{A}}$ . Complexes **4-6** were found by ^31^P NMR spectroscopy studies to contain a mixture of species in solution, one of which crystallised as [Au_3_(tripod|)_2_Cl_2_]Cl which was shown by X-ray diffraction to contain both tetrahedral and linear Au(I), the first example of a Au(I) complex containing such a mixture of geometries. The reaction of [Au_3_ (tripod)Cl_3_] (**1**) with tripod led successfully to the formation of [Au_3_(tripod|)_2_Cl_2_]^+^ and [Au_3_(tripod)_2_Cl_3_]^+^ and [Au_3_(tripod|)_3_Cl]^2+^. The silver(I) complexes, **7**  and **8** appear to contain linear and tetrahedral Ag(I), respectively.


### DIFFERENT COORDINATION MODES OF A TRIPOD PHOSPHINE IN GOLD(I) AND SILVER(I) COMPLEXES

P. Sevillano[1], M.E. García*[1], A. Habtemariam[2], S. Parsons[2] and P.J. Sadler[2]

[1] Department of Inorganic Chemistry, University of Santiago de Compostela
E-15706 Santiago de Compostela, Spain
[2] Department of Chemistry, University of Edinburgh
King's Buildings, West Mains Road, EH9 3JJ Edinburgh, U.K.

### ABSTRACT
The following gold(I) and silver(I) complexes of the tritertiary phosphine 1,1,1-tris(diphenylphosphinomethyl)ethane, *tripod*, have been synthesised: $Au_3$(tripod)$X_3$ [X = Cl(**1**), Br(**2**), I(**3**)]; [$Au_3$(tripod)$_2Cl_2$]Cl (**4**); Au(tripod)X [X = Br(**5**), I(**6**)]; $Ag_3$(tripod)$(NO_3)_3$ (**7**), Ag(tripod)$NO_3$ (**8**). They were characterized by X-ray diffraction (complexes **2**, **3** and **4**), $^{31}$P NMR spectroscopy, electrospray and FAB mass spectrometry and infrared spectroscopy. Complexes **2** and **3** show a linear coordination geometry for Au(I), with relatively short Au-P bond distances. Complex **3** has a Au•••Au intramolecular distance of 3.326 Å, while complex **2** had a short Au•••Au intermolecular interaction of 3.048 Å. Complexes **4-6** were found by $^{31}$P NMR spectroscopy studies to contain a mixture of species in solution, one of which crystallised as [$Au_3$(tripod|)$_2Cl_2$]Cl which was shown by X-ray diffraction to contain both tetrahedral and linear Au(I), the first example of a Au(I) complex containing such a mixture of geometries. The reaction of [$Au_3$(tripod)$Cl_3$] (**1**) with tripod led successfully to the formation of [$Au_3$(tripod)$_2Cl_2$]$^+$ and [$Au_3$(tripod)$_3$Cl]$^{2+}$. The silver(I) complexes, **7** and **8** appear to contain linear and tetrahedral Ag(I), respectively.

### INTRODUCTION
In recent years considerable research has been focused on the chemistry of diphosphines, whereas the chemistry of triphosphines is less well developed. Transition-metal complexes of 1,1,1,-tris(diphenylphosphinomethyl)ethane, *tripod*, in which tripod acts as a tridentate chelating ligand, with fac-octahedral geometry are known,[1-3] and five-coordinate complexes with square-pyramidal[4,5] or trigonal-bipyramidal[6-8] geometries have also been reported.

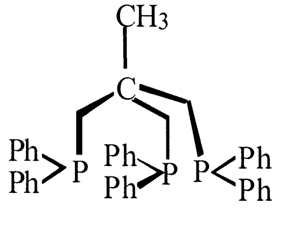

Figure1. Structure of tripod.

Distorted tetrahedral geometries have been found for complexes of the type M(tripod)X [M = Mo, Co, Ni, Pd, Pt, Cu, Ag; X = Ph, NO, $SO_2$, CO, $PR_3$, Cl, I], containing three fused six-membered rings.[9-12] Such complexes can undergo chelate ring-opening reactions.[13] When this occurs the presence of a dangling P atom[14-17] allows further reactions such as oxidation[18,19] or formation of heterobimetallic complexes.[20,21] There is only one example of tripod acting as a tridentate-bridging ligand in which the ligand bridges three chloro-gold(I) fragments and there is a Au•••Au intramolecular interaction.[1]

Our interest in metal phosphine complexes arises from the antiarthritic activity of the linear Au(I) complex auranofin and anticancer activity of tetrahedral Au(I) and Ag(I) diphosphine complexes.[22] We report here the preparation, characterisation and properties of some Au(I) and Ag(I) tripod complexes including a highly unusual Au(I) complex containing both linear and tetrahedral Au(I) centers.

### EXPERIMENTAL
### Materials and Methods
The complexes were prepared using 1,1,1,-tris(diphenylphosphinomethyl)ethane and AuI from Strem Chemicals, $AgNO_3$ from Analema, 2,2 thiodiethanol from Aldrich and metallic gold from Sociedad Española de Metales Preciosos. Solutions of [Au(thiodiglycol)Cl] and [Au(thiodiglycol)Br] were prepared following literature methods.[23,24]

Microanalyses were performed by the University of Santiago de Compostela on a Fisons Instruments EA 1108 CHNS-O. Mass spectra by fast atomic bombardment (FAB) were obtained on a Kratos MS 50 spectrometer using nitrobenzylic alcohol as the matrix. Electrospray mass spectra (ESMS) were recorded on a Micromass VG-QUATTRO spectrometer from 0.5 $10^{-4}$ M solutions of the complexes using $CH_3CN/H_2O$/formic acid 1% as mobile phase. Infrared spectra were recorded at ambient temperature as KBr pellets (4000-500 $cm^{-1}$) and Nujol mulls (500-100$cm^{-1}$) on a Mattson Cygnus 100 spectrophotometer. The bands are reported as vs = very strong, s = strong, m = medium, w = weak, and sh = shoulder. $^{31}P\{^1H\}$ NMR spectra were recorded on a Bruker AMX500 at 202.46 MHz in $CDCl_3$ (room temperature) and $CD_2Cl_2$ (lower temperatures). Chemical shifts are reported in ppm relative to external 85% $H_3PO_4$; (δ= chemical shift in ppm; s = singlet, d = doublet, dd = doublet of doublets, br = broad, J = coupling constant in Hz).

### Crystallography

### Crystal data

Crystal **2**: Empirical formula, $C_{42.33}H_{41.67}Au_3Br_3Cl_{2\,67}P_3$; System, *triclinic*; Space group, *P*-1; $a$ = 13.575(6); $b$ = 18.566(7); $c$ = 20.79(1); $\alpha$ = 67.08(2)°; $\beta$ = 84.72(4)°; $\gamma$ = 82.57(2)°; Vol. = 4781.56 $Å^3$; $Z$ = 4; R =6.37%; parameters 871; ΔF max, min: 2.13, -2.01 e/$Å^3$

Crystal **3**: Empirical formula, $C_{41.5}H_{41}Au_3ClI_3O_{0\,5}P_3$; System, *orthorhombic*; Space group, $Pna2_1$; $a$ =27.866(5); $b$ = 12.358(5); $c$ = 13.575(2); $\alpha$ =90.00°; $\beta$ =90.00°; $\gamma$ =90.00°; Vol. = 46.75(2) $Å^3$; $Z$ = 4; R =7.82%;parameters217;ΔFmax,min:1.68, -1.39 e/$Å^3$

Crystal **4**: Empirical formula, $C_{82}H_{85.1}Au_3Cl_3O_{3\,55}P_6$; System, *triclinic*; Space group, *P*-1; $a$ = 14.694(5); $b$ = 18.931(6); $c$ = 35.091(12); $\alpha$ = 78.541(19)°; $\beta$ = 87.83(3)°; $\gamma$ = 76.24(3)°; Vol. = 9291(6) $Å^3$; $Z$ = 4; R = 9.89%; parameters 645; ΔF max, min: 2.08, -2.82 e/$Å^3$

### Structure determination

Intensity data were collected on a Stoe Stadi-4 diffractometer equipped with an Oxford Cryosystems low-temperature[25] device operating at 220 K. Crystals of **2** and **4** diffracted very weakly and so Cu-Kα radiation was used for data collection on account of its intensity advantage over Mo-Kα radiation, which was used for **3**. All three datasets were collected in ω-θ mode, those for **2** and **3** with on-line profile-fitting.[26] A numerical absorption correction was applied for **2**, the crystal dimensions having been optimised against a set of Ψ-scans;[27] all data for which either the incident or diffracted beams made an angle of less than 1° with the lamina face, (001), were omitted. Absorption corrections for **3** and **4** were based purely on ψ-scans.[28] All structures were solved by Patterson methods (DIRDIF)[29] and completed by iterative cycles of least-squares refinement and difference syntheses (CRYSTALS[30] for **2**; SHELXTS for **3** and **4**).

All the analyses were complicated by the effects of weak diffraction and disorder. In **2** one phenyl group is disordered over two orientations, and this was modelled with two intersecting rigid hexagons. Disordered lattice solvent, assumed to be $CH_2Cl_2$, was treated as described by van der Sluis and Spek,[31] and corresponds to 222 e/cell, which amounts to 1.33$CH_2Cl_2$ per formula unit. All full-weight non-H atoms were refined anisotropically, with H-atoms in calculated positions. In **3** and **4** all the phenyl groups (two of which are disordered in each structure) were refined as rigid bodies, and the light atoms refined isotropically. H-atoms were again placed in calculated positions, although no attempt was made to place H-atoms on water molecules. In **4** charge balance requires two $Cl^-$ per fomula unit; one of these was refined as full weight, the other was disordered over two sites.

### Preparation of compounds

*$Au_3$(tripod)$Cl_3$*, **1**. A solution[23] of [Au(thiodiglycol)Cl] (0.25 g of Au, 0.127 mmol) in MeOH (10 ml), was added dropwise to a solution of tripod (0.2642 g, 0.423 mmol) in $CH_2Cl_2$ (25 ml). The reaction mixture was stirred for 24 h and a white precipitate formed. The solid was filtered off, washed with water and dried *in vacuo*. (Found: C, 36.90; H,. 2.60. Calc. for $C_{41}H_{39}P_3Au_3Cl_3$: C, 37.20; H, 2.90 %). FAB: m/z 1281 (M-Cl, 100%), 1054 (M-2Cl-Au, 11%), 853 (M-2Cl-2Au, 13%).

*$Au_3$(tripod)$Br_3$*, **2**. To a solution of tripod (0.1058 g, 0.1693 mmol) in $CHCl_3$ (20 ml) a solution of [Au(thidiglycol)Br][24] (0.1 g of Au, 0.508 mmol) in $Et_2O$ (30 ml), was added dropwise. The solution was stirred for 24 h at ambient temperature, the volume was reduced and $H_2O$ (30 ml) was added to give a white solid. The solid was filtered off, washed with water, and dried *in vacuo*. Suitable crystals for X-ray diffraction were obtained by recrystallization from $CH_2Cl_2$/MeOH. (Found: C, 33.80; H,. 2.60. Calc. for $C_{41}H_{39}P_3Au_3Br_3$: C, 33.80; H, 2.70 %). FAB: m/z 1370 (M-Br, 9%).

*$Au_3$(tripod)$I_3$*, **3**. To a solution of tripod (0.1 g, 0.16 mmol) in $CH_2Cl_2$ (15 ml), solid AuI (0.1555 g, 0.48 mmol) was added. The suspension was stirred for 1 h under $N_2$ at 0°C. The resultant solution was filtered and n-hexane (75 ml) was added to give a yellow precipitate. The solid was filtered off and dried *in vacuo*. Crystals suitable for X-ray diffraction were obtained from $CH_2Cl_2$/MeOH. (Found: C, 31.40; H,. 2.60. Calc. for $C_{41}H_{39}P_3Au_3I_3$: C, 30.80; H, 2.40 %). FAB: m/z 1469 (M-I, 100%), 1145 (M-2I-Au, 37%), 946 (M-2I-2Au, 10%).

*[Au₃(tripod)₂Cl₂]Cl*, **4**. To a solution of tripod (0.1585 g, 0.254 mmol) in $CHCl_3$ (15 ml) a solution[23] of [Au(thiodiglycol)Cl] (0.05 g of Au, 0.254 mmol) in MeOH (5 ml), was added dropwise. The resultant solution was stirred for 24 h. Afterwards the volume was reduced and $H_2O$ (30 ml) was added to afford a white solid. This was filtered off, washed with water and recrystallized from $CH_2Cl_2/Et_2O$. Crystals suitable for X-ray diffraction were obtained from the same solvents. (Found: C, 51.70; H,. 4.00. Calc. for $C_{41}H_{39}P_3AuCl{\bullet}1.5CH_2Cl_2$: C, 51.80; H, 4.00 %). FAB: m/z (M-2Cl-Au-tripod, 16%), 821 (M-2Cl-2Au-tripod, 20%).

*Au(tripod)Br*, **5**. A solution[24] of [Au(thiodiglycol)Br] (0.0751 g of Au, 0.381 mmol) in $Et_2O$ (30 ml), was added dropwise to a solution of tripod (0.238 g, 0.381 mmol) in $CHCl_3$ (10 ml). The resultant solution was stirred for 24 h and a white precipitate formed. The solid was filtered off, washed with water and dried *in vacuo*. (Found: C, 53.0; H,. 4.00. Calc. for $C_{41}H_{39}P_3AuBr{\bullet}H_2O$: C, 53.60; H, 4.40 %). FAB: m/z 821 (M-Br, 53%).

*Au(tripod)I*, **6**. To a solution of tripod (0.1 g, 0.16 mmol) in $CH_2Cl_2$ (15 ml) solid AuI (0.0519g, 0.16 mmol) was added. The suspension was stirred for 1 h under $N_2$ at 0°C. The resulting solution was filtered off and n-hexane (75 ml) was added. A white solid formed, which was filtered off and dried *in vacuo*. (Found: C, 51.50; H, 4.30. Calc. for $C_{41}H_{39}P_3AuI$: C, 51.90; H, 4.10 %). FAB: m/z 821 (M-I, 53%).

*Ag₃(tripod)(NO₃)₃*, **7**. To a solution of tripod (0.2 g, 0.32 mmol) in acetone (15 ml) a solution of $AgNO_3$ (0.1631 g, 0.96 mmol) in MeOH (15 ml) was added dropwise. The resultant solution was stirred for 72 h, filtered, and the solvent was removed *in vacuo*. The solid residue was recristallized from $CH_2Cl_2$/n-hexane. (Found: C, 42.70; H,. 3.30; N, 3.7. Calc. for $C_{41}H_{39}P_3Ag_3N_3O_9$: C, 43.40; H, 3.40; N, 3.7%). FAB: m/z 1072 (M-$NO_3$, 25%), 902 (M-2$NO_3$-Ag, 41%), 733 (M-3$NO_3$-2Ag, 100%).

*Ag(tripod)NO₃*, **8**. Method a:[32] To a hot solution of tripod (0.2 g, 0.32 mmol) in $CH_3CN$ (5 ml), $AgNO_3$ (0.0544 g, 0.32 mmol) was added as a solid. The mixture was stirred in the dark until it became a clear solution. The final solution was filtered and solvents were slowly evaporated in air. The resultant solid was recrystallized from $CH_2Cl_2$/n-hexane.

Method b: A solution of $AgNO_3$ (0.0544 g, 0.32 mmol) in MeOH (10 ml) was added dropwise to a solution of tripod (0.2 g, 0.32 mmol) in $CH_2Cl_2$ (20 ml). The resulting solution was stirred for 24 h and solvents were evaporated *in vacuo*. The resultant solid was recrystallized from $CH_2Cl_2$/n-hexane. (Found: C, 62.00; H, 4.90; N, 2.3. Calc. for $C_{41}H_{39}P_3AgNO_3$: C, 61.90; H, 4.90; N, 1.8 %). FAB: m/z 733 (M-$NO_3$, 100%).

### RESULTS AND DISCUSSION

### Crystal structures

The crystal structure of **2** (Figure 2, Table I), shows tripod acting as tridentade-bridging ligand. The phosphine bridges three bromo-gold(I) fragments with a short Au•••Au intermolecular interaction of 3.048 Å. Each of the gold atoms has the normal linear coordination geometry with P-Au-Br angles in the range of 168.3-176.5°. The mean Au-P bond length of 2.245 Å is within the expected range, as is the mean Au-Br length of 2.410 Å.[33] An interesting feature of the structure is that two arms of each tripod molecule are crossed almost orthogonally with angles Au-Au-P 87.80, 85.91, 90.46 and 83.71°, which brings the metal atoms into close proximity with intramolcular Au•••Au distances of 3.122 and 3.095 Å. The remaining two arms, one from each ligand of the dimer, are also crossed perpendicularly, with Au-Au-P angles of 91.41 and 100.49°, giving rise to an intermolecular Au•••Au interaction of 3.048 Å, the shortest Au•••Au contact in the dimer.[34] Au•••Au contacts are commonly found in crystal structures of Au(I) complexes[35] and the short distances found for **2** suggest that the interactions are relatively strong. Such interactions in polymeric Au(I) phosphine complexes often give rise to luminiscence behaviour,[36-41] but this has yet to be investigated for **2**.

The iodo-complex **3** has a crystal structure (Figure 3, Table I) similar to the analogous chloride complex[1] but intermolecular interactions are absent. The Au-P distances (2.229-2.261 Å) are within the expected range, as are the Au-I bond lengths (2.548-2.554 Å).[33] Again, two arms of the ligand are crossed orthogonally[42] with Au-Au-P angles of 86.0 and 84.5°, as a consequence of an Au•••Au intramolecular interaction of 3.326 Å.

Table I. Selected bond distances (Å) and angles (°) for **2**, **3** and **4**.

| **2** | | **3** | | **4** | |
|---|---|---|---|---|---|
| P1 - Au1 | 2.248(4) | | | P11 - Au(1) | 2.391(9) |
| P2 - Au2 | 2.246(4) | | | P22 - Au(1) | 2.400(8) |
| P3 - Au3 | 2.245(4) | P1 - Au1 | 2.229(9) | P12 - Au(1) | 2.424(8) |
| P4 - Au4 | 2.249(4) | P2 - Au2 | 2.254(10) | P31 - Au(1) | 2.426(9) |
| P5 - Au5 | 2.241(4) | P3 - Au3 | 2.261(10) | P32 - Au(2) | 2.215(12) |
| P6 - Au6 | 2.241(3) | | | P21 - Au(3) | 2.241(10) |
| Au1 - Br1 | 2.411(2) | | | | |
| Au2 - Br2 | 2.413(2) | Au1 - I1 | 2.548(4) | Au(2) - Cl(2) | 2.292(11) |
| Au3 - Br3 | 2.402(2) | Au2 - I2 | 2.551(3) | Au(3) - Cl(3) | 2.273(10) |
| Au4 - Br4 | 2.420(2) | Au3 - I3 | 2.554(3) | | |
| Au5 - Br5 | 2.410(2) | | | | |
| Au6 - Br6 | 2.395(2) | | | | |
| Au1 - Au4 | 3.048(8) | | | | |
| Au2 - Au3 | 3.122(9) | Au1 - Au2 | 3.326(2) | | |
| Au5 - Au6 | 3.095(8) | | | | |
| P1 - Au1 - Br1 | 171.90(1) | | | | |
| P2 - Au2 - Br2 | 173.70(1) | P1 - Au1 - I1 | 172.20(3) | P21 - Au3 - Cl3 | 177.70(4) |
| P3 - Au3 - Br3 | 174.00(1) | P2 - Au2 - I2 | 172.80(3) | P32 - Au2 - Cl2 | 178.50(3) |
| P4 - Au4 - Br4 | 168.30(1) | P3 - Au3 - I3 | 178.50(3) | | |
| P5 - Au5 - Br5 | 174.00(1) | | | | |
| P6 - Au6 - Br6 | 176.50(1) | | | | |
| Au4 - Au1 - P1 | 91.41(9) | | | P22 -Au1 - P12 | 94.30(3) |
| Au3 - Au2 - P2 | 87.80(1) | | | P11 - Au1 - P31 | 95.40(3) |
| Au2 - Au3 - P3 | 85.91(9) | Au2 - Au1 - P1 | 86.00(2) | P12 - Au1 - P31 | 107.60(3) |
| Au1 - Au4 - P4 | 100.49(9) | Au1 - Au2 - P2 | 84.50(3) | P22 - Au1 - P31 | 116.80(3) |
| Au6 - Au5 - P5 | 90.46(9) | | | P11 - Au1 - P12 | 117.00(3) |
| Au5 - Au6 - P6 | 83.71(9) | | | P11 - Au1 - P22 | 125.90(3) |
| Au4 - Au1 - Br1 | 93.58(5) | | | | |
| Au3 - Au2 - Br2 | 95.79(7) | | | | |
| Au2 - Au3 - Br3 | 99.74(6) | Au2 - Au1 - I1 | 101.74(10) | | |
| Au1 - Au4 - Br4 | 91.04(4) | Au1 - Au2 - I2 | 101.85(11) | | |
| Au6 - Au5 - Br5 | 88.65(5) | | | | |
| Au5 - Au6 - Br6 | 99.67(5) | | | | |

The single-crystal X-ray structures of complexes **2** and **3** confirm that they contain Au:P:halide in a 1:1:1 ratio. However, complex **4**, crystallised from $CH_2Cl_2/Et_2O$ solution as $[Au_3(tripod)_2Cl_2]Cl$, in which Au(I) exhibits both tetrahedral and linear geometries (Figure 4; Table I). This complex appears to be the first example of a cation in which tripod acts both as a bidentate chelating and monodentate bridging ligand and which has been characterised by X-ray crystallography. In the literature there are reports of mixed-valence Au(I)/Au(III) complexes with this ligand[43] and other phosphines,[44-47] in which Au(I) and Au(III) have coordination numbers of two and four, respectively. However, to date no examples of Au(I) complexes with this phosphine, showing both geometries for Au(I) in the same compound, have been reported. The central Au(1) is tetrahedral (Figure 4) being bound to P(1) and P(2) of one tripod ligand and to P(1) and P(3) of another tripod. Au-P distances for tetrahedral Au(I) are ca. 2.4 Å (Table I) and are longer than for linear Au-P, 2.23 Å. The bond lengths for linear Au-Cl, 2.292 Å and 2.273 Å, are similar to those reported for complex **1** by Cooper *et al.*[1] P-Au-P angles of 94.3, 95.4, 107.6, 116.8, 117.0 and 125.9° arise from a distorted tetrahedral geometry imposed by the structure of the ligand and the size of chelate rings. The linear Au(I) have P-Au-Cl angles of 177.7 and 178.5°.

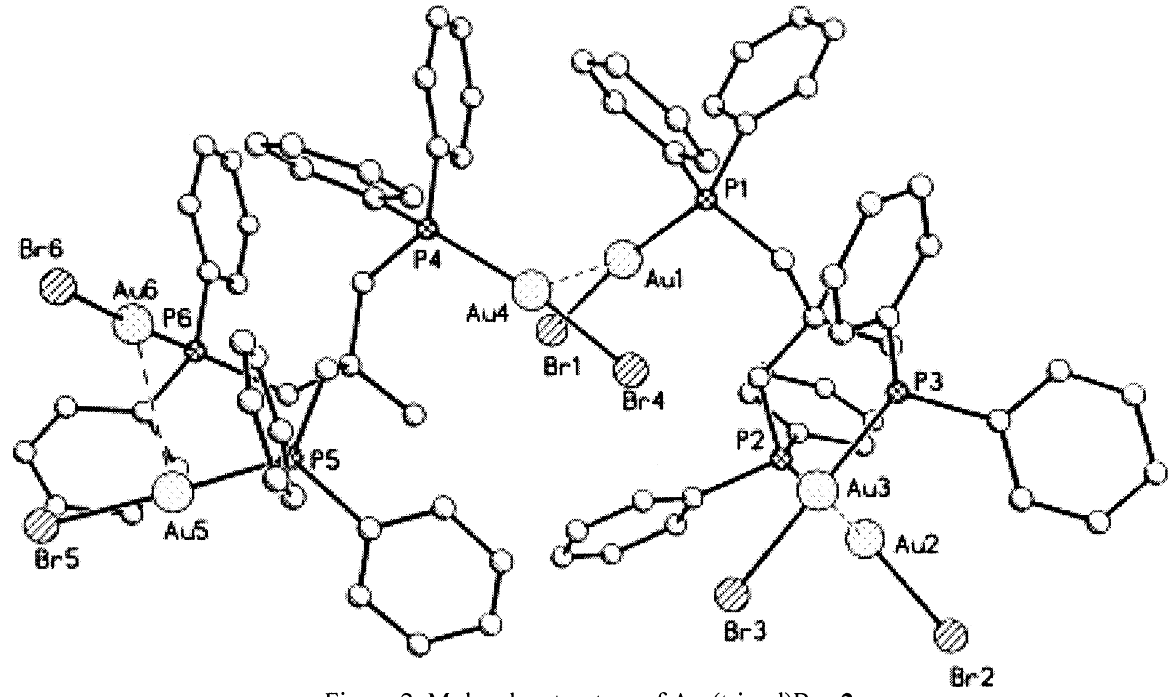

Figure 2. Molecular structure of $Au_3$(tripod)$Br_3$, **2**.

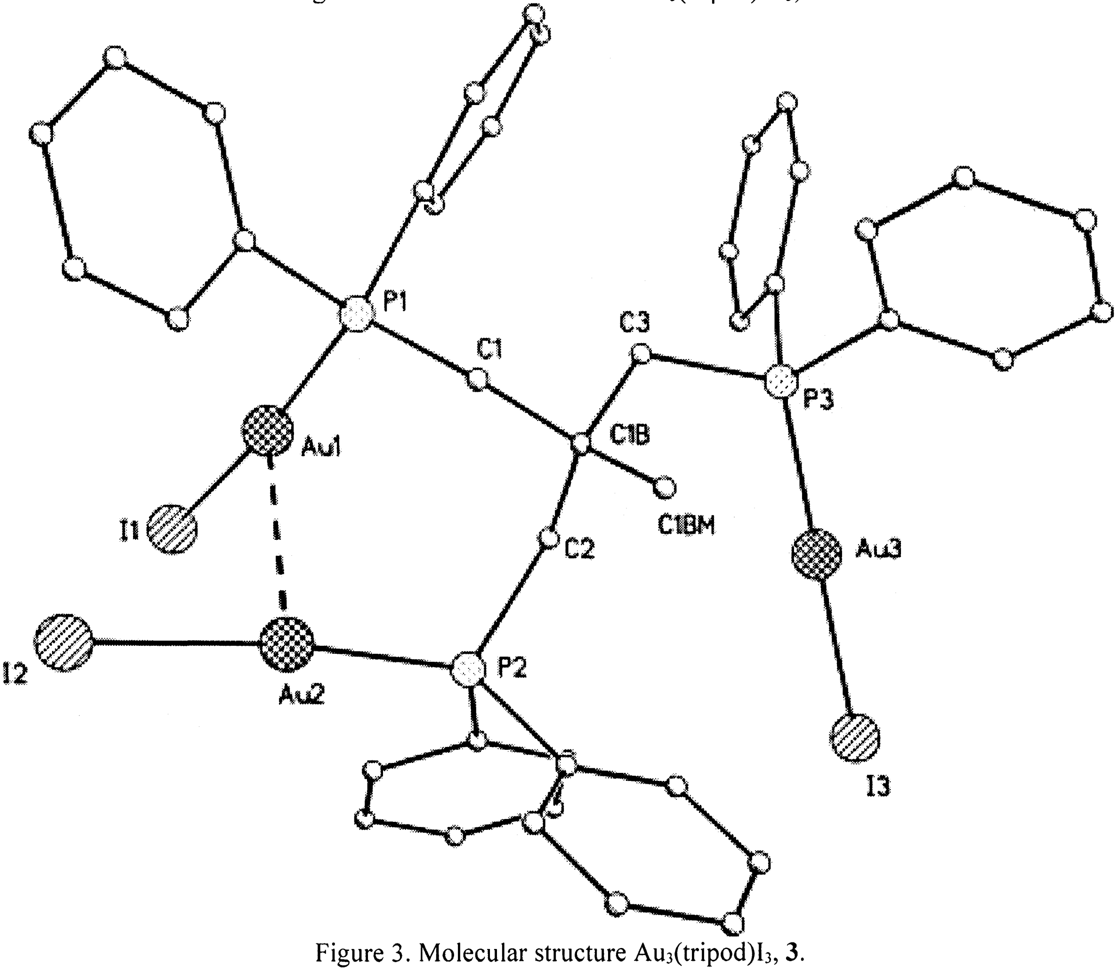

Figure 3. Molecular structure $Au_3$(tripod)$I_3$, **3**.

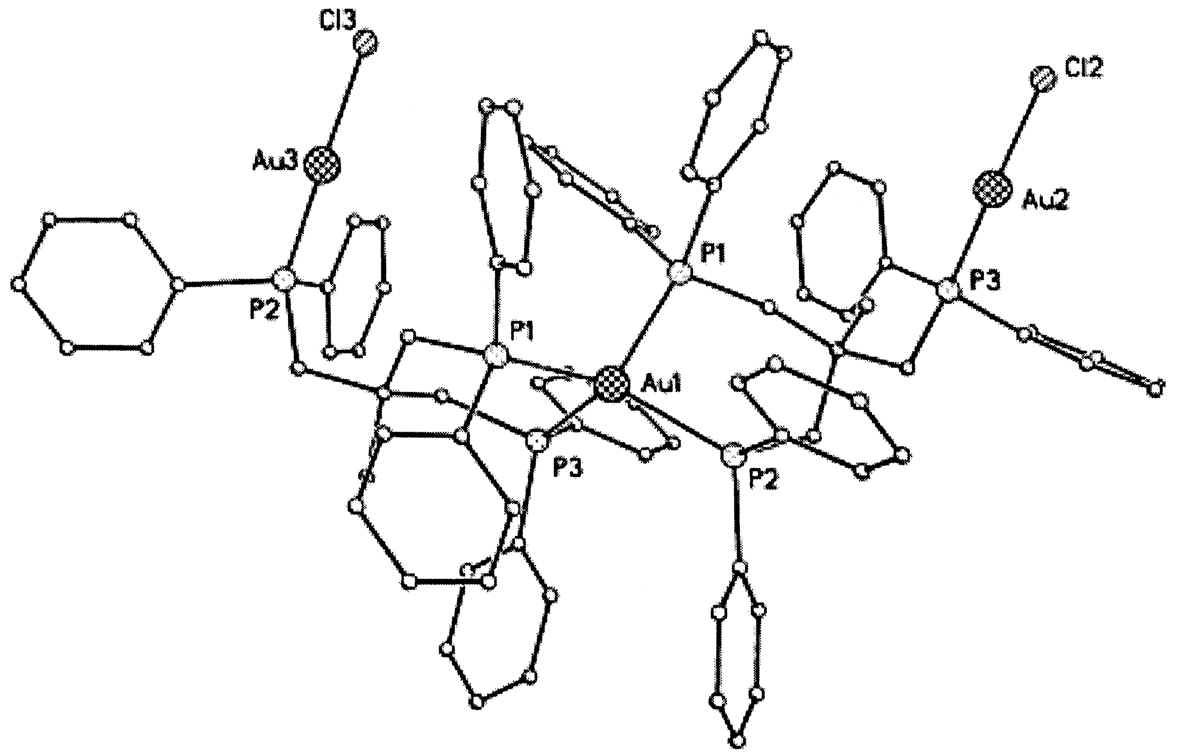

Figure 4. Molecular structure of $[Au_3(tripod)_2Cl_2]Cl$, **4**.

Table II. $^{31}$P NMR ($CDCl_3$ / $CD_2Cl_2$*) and IR spectroscopy data.

| Compound | δ $^{31}$P (ppm) | $^{1}$J(P-Ag) (Hz) | ν(Au-X) ($cm^{-1}$) | ν(N-O) ($cm^{-1}$) |
|---|---|---|---|---|
| **1** | 15.18s | – | 329vs | – |
| **2** | 17.35s | – | 233vs | – |
| **3** | 20.41s | – | 171m | – |
| **4*** | 26.00s, 16.40br, -2.90br, -9.96br | – | – | – |
| **5*** | 24.90s, -3.48br, -10.16br | – | 233sh | – |
| **6*** | 25.00s, -3.71br, -9.81br | – | – | – |
| 7 | -8.10d | 733 694/801*† | – | 1384vs, 1290vs, 839m |
| **8** | -7.10dd, -12.3br | 487/558 | – | 1384vs, 829m |

Spectrum recorded at -90°C.

**$^{31}$P NMR Spectroscopy**

**Gold complexes**

The $^{31}$P NMR spectra (Table II) of complexes **1-3**, show singlet resonances (15.18, 17.35 and 20.41 ppm) whereas those for complexes **4-6** show several broad peaks, suggesting that ligand exchange processes are occurring, similar to those reported for related complexes.[43,48] The downfield shift from 15.18 to 20.41 ppm, from the chloro to the iodo complex **1-3**, is probably due to an increase in the metal-to-ligand back-bonding.[1]

The $^{31}$P NMR spectrum of $[Au_3(tripod)_2Cl_2]Cl$ in $CD_2Cl_2$ at room temperature (Table II) shows a sharp singlet resonance at 26.00 ppm which is assigned to oxidised phosphorus of the ligand, and three broad signals at 16.40, -2.90 and -9.96 ppm which are assigned on the basis of known shifts of related Au(I)-phosphine complexes,[49] assuming that $^{31}$P signals are shifted upfield when the coordination number increases. Thus, the signal at 16.40 ppm corresponds to P coordinated to linear Au(I) and the signals at -

9.96 and -2.90 ppm to P in tetrahedral environment. The broadening suggest that some exchange processes are occurring in solution.[43]

On cooling to -90°C, the broad peak at 16.40 ppm give rise to two singlets (19.61 and 15.35 ppm) and the peak at -2.90 ppm at ambient temperature also gave rise to two broad peaks (-0.18 and -4.35 ppm). The broad peak at -9.96 ppm remains broad, and shifts ca. 3 ppm to high field when the temperature is lowered.

$^{31}P$ NMR spectra in $CD_2Cl_2$ of complexes **5** and **6** (Table II) have two broad peaks at -3.48 and -10.16 ppm (**5**), and -3.71 and -9.81 ppm (**6**) also suggesting the presence of some exchange processes. The spectra of **5** and **6** at -90°C show the appearance of new peaks in the region of 30 to 5 ppm, which are associated with P coordinated to linear Au(I). The two broad P resonances (0 to -15 ppm) associated with P in tetrahedral environment also give rise to several broad peaks indicating the presence of different species in exchange in solution. It seems that bromide and iodide also form complexes containing linear and tetrahedral Au(I), giving rise to complicated equilibrium in solution.

**Silver complexes**

The $^{31}P$ NMR spectra of silver(I) compounds (**7-8**) show the typical pair of doublets due to coupling of $^{31}P$ to both $^{107}Ag$ (51.82% abundance) and $^{109}Ag$ (48.18% abundance)[50] and were well resolved at low temperatures (Table II).

The unresolved doublet of doublets (ambient temperature) at δ -8 ppm for complex **7** (Table II) shows $^1J(^{31}P\text{-}Ag)$ ca. 733 Hz. At lower temperatures a pair of doublets is resolved with $^1J(^{31}P\text{-}^{107/109}Ag)$ = 694/801 Hz, which is consistent with each Ag being coordinated[51] to only one P, and nitrate acting as a monodentate ligand. The equivalence of the three $-CH_2-$ groups in solution is also evident from $^1H$ NMR measurements.

The $^{31}P$ NMR spectrum at ambient temperature for **8** shows (Table II) a doublet of doublets at 7 ppm with coupling constants $^1J(^{31}P\text{-}^{107/109}Ag)$ = 487/558 Hz corresponding to Ag(I) coordinated to two $^{31}P$ atoms (Figure 6). Another broad peak appears at -12.3 ppm in $CDCl_3$ solutions that may be due to tetrahedral Ag(I),[52] with nitrate acting mainly as a bidentate ligand in a polynuclear complex allowing facile ligand redistribution.[48] At lower temperatures, or in $CD_2Cl_2$ solutions, the broad peak disappears.

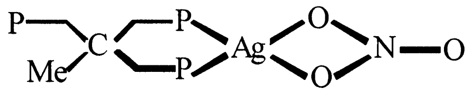

Figure 6. Possible structure of $Ag(tripod)NO_3$

**Titrations**

The complex $Au_3(tripod)Cl_3$, **1**, was titrated with 0.5, 1, 1.5 and 2 mol equivalents of tripod and the course of the reaction was followed by $^{31}P$ NMR spectroscopy (Figure 5). The $^{31}P$ spectrum shows that when 1 mol. equiv. of tripod is added, complex **1** was converted to $[Au_3(tripod)_2Cl_2]^+$ i.e. complex **4**. When 2 mol. equiv. of tripod were added, the product was $[Au_3(tripod)_3Cl]^{2+}$.

**IR Spectroscopy**

The IR data show the presence of terminal Au-X bonds in complexes **1-6** and the coordinated nitrate in compounds **7-8** (Table II).

**Electron Spray Mass Spectrometry (ESMS)**

Ambient temperature ESMS can reveal the individual components of a system in which redistribution and exchange of phosphine ligands is fast on the NMR time scale. Thus ESMS spectra were recorded for complexes **4-6** (Table III) in order to characterise the different species formed in solution.

The strongest peaks in the ESMS spectra of complex **4** are assigned to $[Au_2(tripod)_2]^{2+}$ (m/z 821), $[Au_3(tripod)_2Cl]^{2+}$ (m/z 937) and to the monoxidized complexes $[Au_2(tripod\text{-}O)_2]^{2+}$ (m/z 837) and $[Au(tripod)(tripod\text{-}O)Cl]^{2+}$ (m/z 945). The formation of the phosphine oxide ligands (Table III) is common in the electrospray ion source experiments on phosphine complexes and has been noted previously for other Au(I)-phosphine complexes.[53,54] Thus, the formation of $[Au_3(tripod)_2Cl]^{2+}$ reveals that in solution the parent complex might be $[Au_3(tripod)_2Cl_2]^+$. However, since ESMS does not give direct structural information[55] for determining the coordination mode of tripod further NMR studies were undertaken.

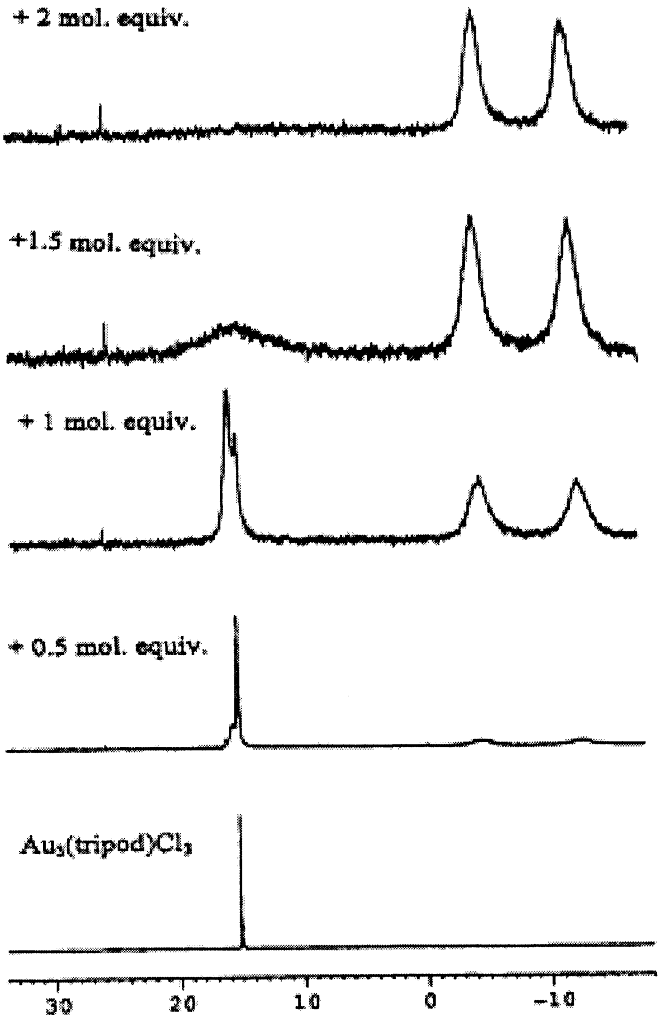

Figure 5. Titration of $Au_3(tripod)Cl_3$ with tripod

Table III. Positive ion ESMS data for complexes **4-6**.

| Complex | m/z | Species |
|---|---|---|
| **4** | 945 | $[Au(tripod)(tripod\text{-}O)Cl]^{2+}$ |
| | 937 | $[Au_3(tripod)_2Cl]^{2+}$ |
| | 837 | $[Au_2(tripod\text{-}O)_2]^{2+}$ |
| | 821 | $[Au_2(tripod)_2]^{2+}$ |
| **5** | 1289 | $[Au_3(tripod)(tripod\text{-}O)_2Br]^{2+}$ |
| | 1281 | $[Au_3(tripod)_2(tripod\text{-}O)Br]^{2+}$ |
| | 1273 | $[Au_3(tripod)_3Br]^{2+}$ |
| | 1150 | $[Au_2(tripod)(tripod\text{-}O)_2]^{2+}$ |
| | 1142 | $[Au_2(tripod)_2(tripod\text{-}O)]^{2+}$ |
| | 1134 | $[Au_2(tripod)_3]^{2+}$ |
| | 822 | $[Au_3(tripod)_3]^{3+}$ |
| **6** | 1312 | $[Au_3(tripod)(tripod\text{-}O)_2I]^{2+}$ |
| | 1304 | $[Au_3(tripod)_2(tripod\text{-}O)I]^{2+}$ |
| | 1296 | $[Au_3(tripod)_3I]^{2+}$ |
| | 1150 | $[Au_2(tripod)(tripod\text{-}O)_2]^{2+}$ |
| | 1142 | $[Au_2(tripod)_2(tripod\text{-}O)]^{2+}$ |
| | 1134 | $[Au_2(tripod)_3]^{2+}$ |
| | 822 | $[Au_3(tripod)_3]^{3+}$ |

Thus, based on $^{31}P$ NMR spectrum, two possible structures with two different "$AuP_4$" environments can be proposed (Figure 7).

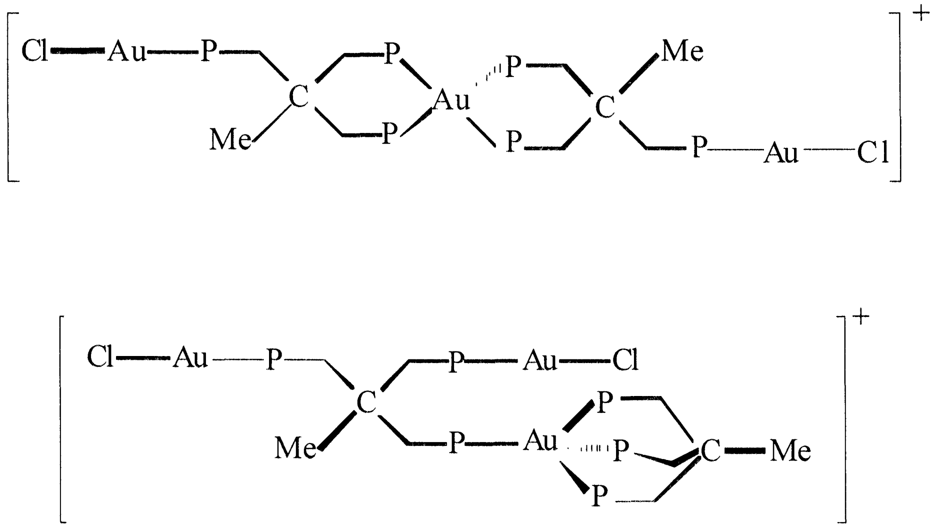

Figure 7. Possible "$AuP_4$" environments in solution for polynuclear species $[Au_3(tripod)_2Cl_2]^+$. Complexes **5** and **6** show the same ESMS spectra with peaks assigned to $[Au_3(tripod)_3X]^{2+}$, $[Au_2(tripod)_3]^{2+}$, $[Au_3(tripod)_3]^{3+}$ and their respective oxides.

**ACKNOWLEDGEMENTS**
We thank Xunta de Galicia (XUGA 20906A98), BBSRC and EPSRC for their support for this work.

**REFERENCES**

1. Cooper, M.K.; Henrick, K.; McPartlin, M.; Latten, J.L. *Inorg. Chim. Acta*, **65**, L185-L186 (1982).
2. Cecconi, F.; Midollini, S.; Orlandini, A.; Sacconi, L. *Inorg. Chim. Acta*, **42**, 59-63 (1980).
3. Ellerman, J.; Lindner, H.A.; Moll, M. *Chem. Ber.*, **112**, 3441-3452 (1979).
4. Ghilardi, C.A.; Laschi, F.; Midollini, S.; Orlandini, A.; Scapacci, G.; Zanello, P. *J. Chem. Soc., Dalton Trans.*, 531-540 (1995).
5. Benelli, C.; Di Vaira, M.; Noccioli, G.; Sacconi, L. *Inorg. Chem.*, **16**,182-187 (1977).
6. Dapporto, P.; Midollini, S.; Orlandini, A.; Sacconi, L. *Inorg. Chem.*, **15**, 2768-2774 (1976).
7. Bianchini, C.; Masi, D.; Mealli, C.; Meli, A. *Inorg. Chem.*, **23**, 2838-2844 (1984).
8. Janser, P.; Venanzi, L.M.; Bachechi, F. *J. Organomet. Chem.*, **296**, 229-242 (1985).
9. Kowalski, A.S.; Ashby, M.T. *J. Am. Chem. Soc.*, **117**, 12639-12640 (1995).
10. Vogel, S.; Huttner, G.; Zsolnai, L.; Emmerich, C. *Z. Naturforsch*, **B48**, 353-363 (1993).
11. Grevin, J.; Kalck, P.H.; Daran, J.C.; Vaissermann, J.; Bianchini, C. *Inorg. Chem.*, **32**, 4965-4967 (1976).
12. Mason, R.; Williams, G.A. *Aust. J. Chem.*, **34**, 471-477 (1981).
13. Sevillano, P.; García, M.E.; Parsons, S.; Habtemariam, A.; Sadler, P.J. unpublished work.
14. Chatt, J.; Leigh, G.J.; Jhankarajan, N. *J. Organomet. Chem.*, **29**, 105-110 (1971).

15. Brandt, K.; Sheldrick, W.S. *Chem. Ber.*, **129**, 1199-1206 (1996).
16. Blake, A.J.; Gould, R.O.; Halcrow, M.A.; Schroder, M. *J. Chem. Soc., Dalton Trans.*, 2909-2920 (1993).
17. Kirchner, R.M.; Little, R.G.; Tan, K.D.; Meek, D.W. *J. Organomet. Chem.*, **149**, C15-C18 (1978).
18. Habtemariam, A.; Sadler, P.J.; Castiñeiras, A.; Sevillano, P.; García, M.E. XXXII International Conference on Coordination Chemistry, Santiago de Chile, Chile, 5P36 (1997).
19. Sevillano, P.; Habtemariam, A.; Castiñeiras, A.; García, M.E.; Sadler, P.J. *Polyhedron*, **18**, 383-389 (1998).
20. Fernández, E.J.; Gimeno, M.C.; Jones, P.G.; Laguna, A.; Laguna, M.; Olmos, E. *J. Chem. Soc., Dalton Trans*, 3603-3608 (1996).
21. Sevillano, P.; García, M.E.; Parsons, S.; Habtemariam, A.; Sadler, P.J. XXXIII International Conference on Coordination Chemistry, Florence, Italy, 540T (1998).
22. Berners-Price, S.J.; Sadler, P.J. *Struct. Bond.*, **70**, 227, (1988)
23. Baddley, W.H.; Basolo, F.; Gray, H.B.; Nölting, C.; Poë, A.J. *Inorg. Chem.*, **2**, 921-928 (1963).
24. Gregory, B.J.; Ingold, C.K. *J. Chem. Soc., Sec. B*, 276-289 (1969).
25. Casier, J.; Glaze, A.M. *J. Appl. Cryst.* **19**, 205 (1986).
26. Clegg, W. *Acta Cryst.*, **A37**, 22 (1981).
27. StoeXShape, Stoe and Ceie, Darmstadt (1996).
28. Sheldrick, G.M. Shelxtl V.S., Scemens Analytical X-ray instruments, Madison, Wisconsin (1995).
29. Beurskens, P.T.; Beurskens, G.; Bosman, W.P.; de Gelder, R.; García-Granda, S.; Gould, R.O.; Israël, R.; Smits, J.M.M. DIRDIF-96, Crystallography Laboratory, University of Nijnegen, Holland.
30. Watkin, D.J.; Prout, C.K.; Betteridge, P.W.; Curruthers, J.R. CRYSTALS, Issue 10, Chemical Crystallography Laboratory, University of Oxford (1996).
31. van der Sluis, P.; Spek, T. *Acta Cryst.*, **A46**, 194, (1990).
32. Camalli, M.; Caruso, F. *Inorg. Chim. Acta*, **169**, 189-194 (1990)
33. Orpen, A.G.; Brammer, L.; Allen, F.H.; Kennard, O.; Watson, D.G.; Taylor, R. *J. Chem. Soc., Dalton Trans.*, S1-S83 (1989).
34. Stützer, A.; Bissinger, P.; Schmidbaur, H. *Z. Naturforsch*, **47b**, 1261-1266 (1992).
35 Schmidbaur, H.; Pollok, Th.; Herr, R.; Wagner, F.E.; Bau, R.; Riede, J.; Müller, G. *Organomet.*, **5**, 566 (1986)
36. Berning, D.E.; Katti, K.V.; Barnes, Ch. L.; Volkert, W.A.; Ketring, A.R. *Inorg. Chem.*, **36**, 2765-2769 (1997).
37. Forward, J.M.; Bohmann, D.; Fackler, Jr., J.P.; Staples, R.J. *Inorg. Chem.*, **34**, 6330-6336 (1995).
38. King, C.; Wang, J.C.; Khan, Md.N.I.; Fackler, Jr., J.P. *Inorg. Chem.*, **28**, 2145-2149 (1989).

39. Assefa, Z.; McBurnett, B.G.; Staples, R.J.; Fackler, Jr., J.P. *Inorg. Chem.*, **34**, 4965-4972 (1995).
40. Che, Ch.-M.; Kwong, H.L.; Poon, Ch.-K. *J. Chem. Soc., Dalton Trans.*, 3215-3219 (1990).
41. McCleskey, T.M.; Gray, H.B. *Inorg. Chem.*, **31**, 1734-1740 (1992).
42. Grohmann, A.; Schmidbaur, H. *Comprehensive Organomet. Chem.*, Oxford Press, Vol 3 (1995).
43. Fernández, E.J.; Gimeno, M.C.; Laguna, A.; Laguna, M.; López-de-Luzuriaga, J.M.; Olmos, E. *J. Organomet. Chem.*, **514**, 169-175 (1996).
44. Raptis, R.G.; Porter, L.C.; Emrich, R.J.; Murray, H.H.; Fackler, Jr., J.P. *Inorg. Chem*, **29**, 4408-4412 (1990)
45. Fernández, E.J.; Gimeno, M.C.; Jones, P.G.; Ahrens, B.; Laguna, A.; Laguna, M.; López-de-Luzuriaga, J.M. *J. Chem. Soc., Dalton Trans.*, 3487-3492 (1994).
46. Usón, R.; Laguna, A.; Laguna, M.; Manzano, B.R.; Jones, P.G.; Sheldrick, G.M. *J. Chem. Soc., Dalton Trans.*, 839-843 (1984).
47. Fernández, E.J.; Gimeno, M.C.; Jones, P.G.; Laguna, A.; Laguna, M.; López-de-Luzuriaga, J.M. *J. Chem. Soc., Dalton Trans.*, 3365-3370 (1992).
48. Papathanasion, P.; Salem, G.; Waring, P.; Willis, A.C. *J. Chem. Soc., Dalton Trans.*, 3435-3443 (1997).
49. Sevillano, P.; García, M.E.; Harvey, P.; Berners-Price, S.J.; Habtemariam, A.; Sadler, P.J. 3rd GIPS Meeting in Inorganic Chemistry, Senigallia, Italy (1995), P34.
50. Mann, B.E. "NMR in Inorganic Chemistry", Encyclopedia of Inorganic Chemistry, John Wyley & Sons, Chichester, England, **7**, 2615-2660 (1994).
51. Banon, P.F.; Dyason, J.C.; Healy, P.C.; Engelhardt, L.M.; Skelton, B.W.; White, A.H. *J. Chem. Soc., Dalton Trans.*, 1965-1970 (1986).
52. Affandi, D.; Berners-Price, S.J.; Effendy; Harvey, P.J.; Healy, P.C.; Ruch, B.E.; White, A.H. *J. Chem. Soc., Dalton Trans.*, 1411-1420 (1997).
53. Colton, R.; James, B.D.; Potter, I.D.; Traeger, J.C. *Inorg. Chem.*, **32**, 2626-2629 (1993).
54. Bond, A.M.; Colton, R.; Traeger, J.C.; Harvey, J. *Inorg. Chim. Acta*, **228**, 193-197 (1995).
55. Colton, R.; Harrison, K.L.; Mah, Y.A.; Traeger, J.C. *Inorg. Chim. Acta*, **231**, 65-71 (1995).

**Received: October 3, 1998 - Accepted in final form: April 22, 1999**

